# Perceived sustainability authenticity as a place-based psychological cue: moral emotions, psychological ownership, and responsible environmental cooperation in nature-based accommodation

**DOI:** 10.3389/fpsyg.2026.1903093

**Published:** 2026-07-14

**Authors:** Lin Sun, Juan Li, Muhammad Arif

**Affiliations:** 1 School of History, Culture, and Tourism, Guangxi Normal University, Guilin, China; 2 Guilin Zhuoran School, Guilin, China; 3 School of Economics and Business Administration, Heilongjiang University, Harbin, China

**Keywords:** green skepticism, moral emotions, nature-based accommodation, perceived sustainability authenticity, psychological ownership, responsible environmental cooperation, soft behavioral governance

## Abstract

Tourism sustainability partly depends on guests’ voluntary cooperation. However, how the perceived authenticity of place-based environmental practices relates to discretionary conduct during an accommodation stay remains insufficiently understood. Here, we examined whether perceived sustainability authenticity was associated with self-reported responsible environmental cooperation through moral emotions and psychological ownership and whether these indirect associations varied with green skepticism. We surveyed 337 guests staying in rural nature-based accommodation in Yangshuo, China. Covariance-based structural equation modelling was used to evaluate the measurement and structural models, and bootstrapping was used to estimate indirect and conditional indirect associations. Perceived sustainability authenticity was positively associated with responsible environmental cooperation, both directly and indirectly through moral emotions and psychological ownership. The two indirect associations were consistent with conceptually distinct affective and responsibility-oriented pathways. Both were larger among guests with higher green skepticism, and the corresponding indices of moderated mediation excluded zero. This pattern was consistent with the interpretation that skeptical guests applied more stringent credibility assessments when comparing environmental claims with observable practices. The findings position perceived sustainability authenticity as a contextual credibility cue associated with voluntary environmental cooperation and distinguish the psychological meanings of moral emotions and psychological ownership. Because the data were cross-sectional, single-source, and self-reported, they did not establish temporal ordering, causal mediation, objectively verified environmental conduct, or persistence beyond the accommodation stay. Even so, the findings point to a clear practical priority: sustainability claims need to be matched by visible routine practices, while guest participation remains voluntary, credible, and minimally burdensome.

## 1 Introduction

Tourism systems are closely coupled with environmental resources on which destination attractiveness and visitor experiences depend (Orea-Giner et al., 2025; Tian et al., 2026). Accommodation providers have increasingly adopted energy-efficient technologies, water-conservation measures, waste-reduction programs, and environmental certification schemes in response to growing ecological pressures and institutional expectations (Mo et al., 2025; Nguyen et al., 2025). The scale of the underlying challenges remains substantial. The United Nations Environment Programme estimated that food-service activities accounted for 28% of the 1.05 billion tons of food wasted globally in 2022 (Devaraj and Balasubramanian, 2025). Organizational initiatives alone, however, do not encompass all environmentally relevant activity occurring during an accommodation stay. Resource conservation, waste separation, and compliance with environmental arrangements are also partly contingent on guests’ voluntary participation. Such cooperation cannot be presumed, particularly when responsible conduct entails additional effort, reduced convenience, or personal sacrifice (Li et al., 2026; Liu et al., 2025; Thai and Nguyen, 2022). A central practical problem for sustainable accommodation is therefore the discrepancy between the availability of environmental initiatives and guests’ willingness to support them through discretionary conduct.

Tourism research has addressed this problem largely through green communication, eco-labels, informational appeals, and models of environmental attitudes and behavioral intentions (Iftikhar et al., 2024; Manosuthi et al., 2026; Wang et al., 2023). Related studies have examined how sustainability information corresponds to awareness, evaluations, and stated behavioral preferences (Yu et al., 2025). This literature has provided important insights into tourists’ cognitive responses to environmental communications (Ciki and Tanriverdi, 2024). Nevertheless, favorable attitudes and intentions do not necessarily correspond with discretionary conduct during a stay, particularly where environmental actions impose immediate individual costs while their benefits are diffused and collectively shared. Communication-centered approaches also tend to conceptualize sustainability as information conveyed by an organization and evaluated by a consumer. They offer less insight into guests’ post-arrival interpretations, when environmental claims can be assessed against routine operations, physical arrangements, and staff practices. A full account of within-stay cooperation therefore requires attention not only to the content of sustainability communication but also to the perceived credibility of the practices encountered.

Perceived sustainability authenticity captures this credibility assessment. It refers to guests’ judgments that environmental practices are genuine, transparent, internally consistent, and embedded in ordinary operations rather than staged primarily for promotional purposes (Chua et al., 2024; Moon et al., 2023; Yu et al., 2024). Sustainability practices can accordingly be interpreted as place-based psychological cues encountered through the material and social organization of an accommodation experience. Guests may compare environmental claims with observable practices and assess whether the two correspond. Perceived discrepancies can be associated with inferences of exaggeration, symbolic environmentalism, or greenwashing, whereas visible consistency can correspond with greater legitimacy and normative relevance (Alyahia et al., 2024; Pham et al., 2025). This study interprets such relationships through a soft behavioral governance lens. In contrast to regulatory or sanction-based governance, soft behavioral governance concerns contextual and normative signals associated with voluntary alignment rather than compulsory compliance (Lin et al., 2023). The lens is used as a bounded interpretive framework for organizing the proposed associations; it is not presented as a new theory or as evidence that contextual cues determine guest conduct.

Perceived credibility alone does not fully specify the psychological meanings associated with environmental cooperation. Two conceptually distinct responses are relevant. Moral emotions are affective and norm-responsive reactions, including inspiration, pride, and elevation, associated with the appraisal of conduct as ethically appropriate or beneficial to collective welfare (Al-Atwi et al., 2024; Casado-Diaz et al., 2020; Walters et al., 2021). Sustainability practices perceived as authentic may correspond with stronger moral emotions because guests can interpret them as credible expressions of environmental responsibility. Psychological ownership represents a different form of personal engagement. It refers to a self-referential sense that a target is psychologically “mine,” accompanied by personal connection, concern, and perceived responsibility despite the absence of legal possession (Li et al., 2021; Wu et al., 2025). Within an accommodation stay, a temporary sense of ownership may be positively related to a willingness to protect shared resources, tolerate inconvenience, and support collective environmental objectives (Chen and Wu, 2024; Yoon et al., 2025). Moral emotions thus concern the affective significance assigned to environmental responsibility, whereas psychological ownership concerns the extent to which caring for the place is experienced as personally relevant. Their parallel specification reflects this conceptual distinction but does not imply statistical independence, temporal simultaneity, or a verified sequence of psychological events.

Guests may also differ in how critically they evaluate sustainability information. “Green skepticism” refers to a tendency to question the sincerity, credibility, or effectiveness of environmental claims (Yoon and Chen, 2017; Zhang et al., 2021). Although skepticism is often associated with less favorable evaluations, it may also function as a credibility filter. Guests with higher skepticism may apply more demanding evidentiary standards when comparing environmental communication to observable practices. Under such conditions, perceived authenticity may be more strongly related to psychological and behavioral responses once claims are judged credible (Bakari et al., 2024). Yangshuo, China, provides a theoretically informative setting for examining these relationships. Its rural accommodation sector is embedded within an ecologically sensitive karst landscape and is frequently positioned around nature, locality, and environmental harmony (Deng et al., 2025; Zhang et al., 2025). The coexistence of landscape dependence, tourism commercialization, and visible accommodation operations provides opportunities for guests to compare sustainability claims with practices encountered during their stay. Yangshuo is therefore treated as an analytically revealing setting for credibility assessment rather than as representative of all rural destinations, accommodation markets, or Chinese tourists.

Taken together, the literature leaves one cumulative gap (Supplementary Table S1): existing research has not sufficiently specified how the perceived authenticity of place-based sustainability practices is concurrently associated with guests’ discretionary environmental cooperation through distinct affective and responsibility-oriented pathways, or whether the magnitude of these indirect associations varies across levels of green skepticism. The study addressed the following research questions: (1) How is perceived sustainability authenticity associated with tourists’ reported within-stay responsible environmental cooperation in nature-based accommodation? (2) Do moral emotions and psychological ownership statistically account for theoretically distinct indirect associations between perceived sustainability authenticity and responsible environmental cooperation? (3) Do the indirect associations through moral emotions and psychological ownership vary across levels of green skepticism? The study examined these questions using cross-sectional survey data from guests staying in rural nature-based accommodation.

## 2 Literature review and hypothesis development

### 2.1 Soft behavioral governance and perceived sustainability authenticity

Soft behavioral governance is treated as an interpretive lens rather than a discrete or newly-formulated theory. The term brings together principles from non-coercive governance and behavioral public policy, where voluntary alignment is associated with contextual signals, perceived legitimacy, and normative meaning rather than formal regulation, material sanctions, or direct enforcement (Tian et al., 2026). In accommodation settings, environmental practices communicate what an organization regards as credible, appropriate, and collectively valued. Their behavioral relevance, therefore, rests not merely on their presence but on how guests interpret their consistency, legitimacy, and correspondence with the service environment (Lin et al., 2026; Mariscal et al., 2026). This lens differs from green marketing, which communicates environmental attributes primarily in relation to consumer evaluations and choices. It also differs from conventional persuasion, which relies on informational or argumentative appeals. It also differs from nudging, where the choice architecture is modified without necessarily requiring a deliberate credibility assessment. Norm-activation theory, self-determination theory, and value–belief–norm theory offer adjacent accounts of moral obligation, internalized motivation, and value-consistent conduct. These theories provide a conceptual background but are not tested in their entirety. Soft behavioral governance is instead used as a bounded framework for interpreting how visible, place-based environmental practices may correspond to voluntary cooperation when guests regard them as legitimate and credible. Perceived sustainability authenticity refers to guests’ judgments that environmental practices are genuine, transparent, operationally consistent, and substantively enacted rather than primarily symbolic or promotional (Alyahia et al., 2024; Chua et al., 2024; Moon et al., 2023). Guests can assess whether environmental claims correspond to routine operations, physical arrangements, and observable service practices. Perceived inconsistent may be associated with exaggeration or greenwashing, whereas a correspondence between claims and practice may be associated with greater credibility and normative relevance (Pham et al., 2025). Authenticity is therefore conceptualized as perceived contextual cue rather than verified evidence of an accommodation provider’s objective environmental performance.

### 2.2 Responsible environmental cooperation

**Figure 1:**
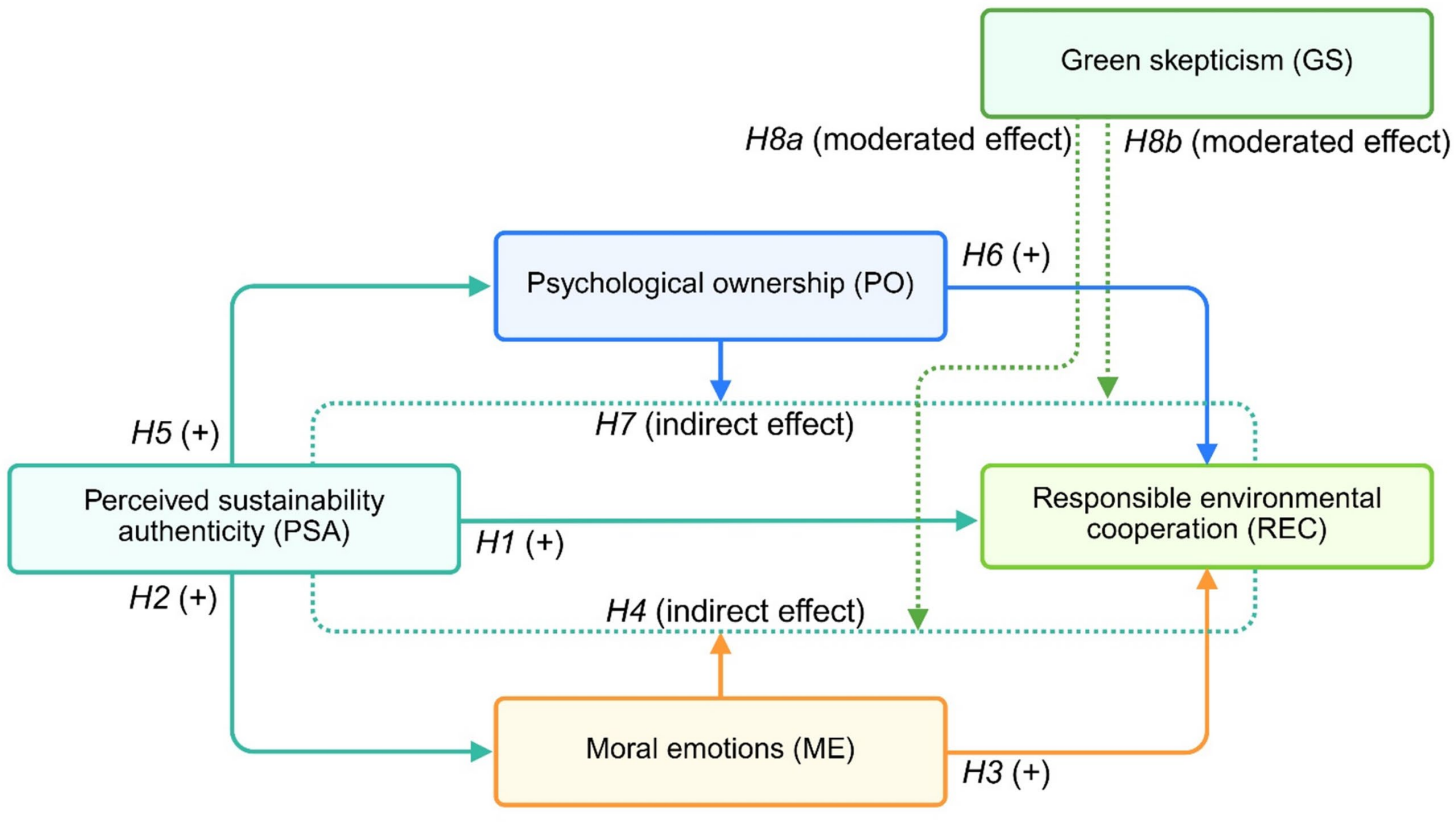
Conceptual framework for sustainability authenticity and responsible tourist behavior.

Responsible environmental cooperation refers to guests’ self-reported discretionary support for environmental objectives during their accommodation stay. It comprises voluntary compliance with environmental practices, willingness to expend additional effort, acceptance of minor inconveniences, encouragement of environmentally responsible conduct among other guests, and constructive support for organizational environmental initiatives (Ai et al., 2025). This operationalization overlaps with, but is not equivalent to, objectively observed pro-environmental behavior, behavioral intention, or customer citizenship behavior (Wang et al., 2023; Xu et al., 2026). The construct is consequently interpreted as a composite of within-staying behavioral cooperation and stated readiness to support environmental practices, not as verified reductions in resource consumption or environmental degradation. This distinction is important because favorable environmental attitudes do not invariably correspond with enacted conduct, particularly when cooperation requires effort, restricts convenience, or generates benefits that are collectively shared. Evidence from nature-based tourism has similarly linked ecologically responsible attitudes with self-determined motivations and subjective well-being, while leaving actual behavioral enactment analytically distinct (Ciki and Tanriverdi, 2024). Under such conditions, perceived authenticity may be especially relevant because discretionary cooperation depends partly on whether the environmental requests appear credible and worthy of voluntary support. Practices perceived as observable, internally consistent, and embedded in ordinary operations are therefore expected to be positively associated with responsible environmental cooperation (Figure 1).

*H1:* Perceived sustainability authenticity was positively associated with responsible environmental cooperation.

### 2.3 Moral emotions as an affective normative pathway

Moral emotions are affective and evaluative responses associated with appraisals of conduct as ethically appropriate or beneficial to collective welfare. Moral inspiration, pride and elevation can render normative considerations emotionally salient, although their presence does not establish that emotional responses temporally precede behavior (Al-Atwi et al., 2024; Casado-Diaz et al., 2020; Walters et al., 2021). Environmental practices perceived as authentic may positively associate with moral emotions because they communicate that environmental responsibility is genuinely valued rather than merely advertised. Moral emotions may, in turn, correspond with responsible cooperation by connecting environmental conduct with an affective appraisal of what is ethically appropriate during the accommodation experience (Baah and Kim, 2025; Li et al., 2021).

*H2:* Perceived sustainability authenticity was positively associated with moral emotions.

*H3*: Moral emotions were positively associated with responsible environmental cooperation.

*H4:* Moral emotions statistically accounted for a positive indirect association between perceived sustainability authenticity and responsible environmental behavior.

### 2.4 Psychological ownership as a responsibility-oriented pathway

Psychological ownership is a self-referential state in which individuals experience a target as psychologically “mine” despite the absence of legal possession. It encompasses personal connection, concern and perceived responsibility and is conceptually distinct from enduring place attachment or long-term identification (Wu et al., 2025). Psychological ownership refers to a temporary, stay-level orientation toward the accommodation and its immediate environment. Environmental practices perceived as authentic and integrated into the guest experience may be positively related to psychological ownership because they present environmental care as a credible and shared responsibility. Guests who regard an accommodation or its environmental condition as partly “theirs” may report greater readiness to protect shared resources, tolerate minor inconveniences and support collective environmental objectives (Chen and Wu, 2024; Yoon et al., 2025).

*H5:* Perceived sustainability authenticity was positively associated with psychological ownership.

*H6:* Psychological ownership is positively associated with responsible environmental cooperation.

*H7:* Psychological ownership statistically accounted for a positive indirect association between perceived sustainability authenticity and responsible environmental cooperation.

### 2.5 Distinction and parallel specification of the pathways

Moral emotions and psychological ownership represent conceptually distinct but potentially correlated responses. Moral emotions are affective and norm-responsive, capturing the extent to which guests feel ethically moved by credible environmental practices (Tran et al., 2025; Zhang et al., 2025). Psychological ownership is self-referential and possession-related, capturing the extent to which guests regard the accommodation or its environmental condition as partly “mine” or “my responsibility.” Their parallel specification enables the simultaneous estimation of two theoretically differentiated indirect associations. It does not establish that the responses are statistically independent, temporally simultaneous or ordered in the proposed direction. Given the cross-sectional design, reciprocal relationships and alternative temporal sequences remain plausible.

### 2.6 Green skepticism as a conditional credibility filter

Green skepticism refers to a tendency to question the sincerity, credibility or environmental effectiveness of organizational claims (Yoon and Chen, 2017; Zhang et al., 2021). Although skepticism is commonly associated with less favorable evaluations, it may also represent a more demanding standard of evidentiary assessment. Guests with higher skepticism may attend more closely to whether environmental communication corresponds with observable practices. Where such correspondence is limited, skepticism may be associated with less favorable responses; where practices appear genuine and operationally embedded, perceived authenticity may become more strongly related to moral emotions, psychological ownership and responsible environmental cooperation (Bakari et al., 2024; Rahman et al., 2015). Green skepticism is therefore conceptualized as a conditional credibility filter associated with variation in the two indirect relationships. This specification concerns differences in the magnitude of statistical associations across levels of skepticism. It does not establish that skepticism changes an underlying causal or temporal process.

*H8a:* The positive indirect association between perceived sustainability authenticity and responsible environmental cooperation through moral emotions was stronger at higher levels of green skepticism.

*H8b:* The positive indirect association between perceived sustainability authenticity and responsible environmental cooperation through psychological ownership was stronger at higher levels of green skepticism.

## 3 Materials and methods

### 3.1 Research design and analytical strategy

The study used a quantitative, cross-sectional, single-wave survey design to examine concurrent associations among perceived sustainability authenticity, moral emotions, psychological ownership, responsible environmental cooperation, and green skepticism. An on-site questionnaire was used because the focal constructs concerned guests’ evaluations, affective responses, self-referential perceptions, and self-reported environmental cooperation during an accommodation stay. Comparable survey designs have been used to examine latent psychological associations in tourism and hospitality settings (Meng et al., 2022; Yu et al., 2025). Covariance-based structural equation modeling (CB-SEM) was used to evaluate the measurement and structural models. This approach was consistent with the theory-testing objective, the use of latent constructs, and the requirement to account for measurement errors while estimating several associations simultaneously. Direct and indirect associations were estimated within the structural model, followed by conditional process analysis across all levels of green skepticism. Because all variables were measured at one time point, the analysis represented contemporaneous covariance patterns. It did not establish temporal ordering, causal mediation, or the direction of the reported relationships.

### 3.2 Research context

The study was conducted in rural hotels in Yangshuo County, Guilin, China (24°46′42.5″N, 110°29′47.7″E). Yangshuo is characterized by an ecologically sensitive karst landscape and a substantial dependence on nature-based tourism (Deng et al., 2025). Rural accommodation is embedded within this visible landscape, where environmental quality forms part of both the visitor experience and the commercial positioning of hospitality businesses. Hotels commonly refer to environmental harmony, local authenticity, and sustainability in their service design and promotional communication (Tian et al., 2026). Yangshuo was theoretically informative because guests could compare environmental claims with observable hotel operations. This included waste management arrangements, energy-saving facilities, service routines, and the physical integration of accommodation into the surrounding landscape. The coexistence of tourism commercialization and conservation concerns also provided conditions in which guests could assess whether environmental commitments appeared substantive or symbolic (Ai et al., 2025; Arif and Yu, 2026). These characteristics were relevant to perceived sustainability and authenticity and to the proposition that skeptical guests might apply more demanding credibility assessments. Yangshuo was therefore treated as an analytically relevant setting rather than representative of all rural destinations, accommodation providers, or tourists.

### 3.3 Sampling and data collection

Data were collected during 2024 over several weeks from March to June across multiple participating rural hotels in Yangshuo. Hotels were included when they were situated in rural or nature-based areas and publicly communicated identifiable environmental practices, including waste-reduction arrangements, energy-saving systems, or environmentally-oriented service designs. On-site intercept convenience sampling was used because access to guests was bounded by the accommodation setting and eligibility required a direct experience of the focal service environment (Sun and Kim, 2024). Guests were approached in the lobbies and shared public areas after completing at least one overnight stay. Eligible respondents were current guests who could independently complete the questionnaire and assess environmental practices encountered during their stay. The overnight-stay criterion provided respondents with direct exposure to hotel operations before participation (Gong et al., 2025). The shortest-stay category reported which denotes one completed overnight stay. Participation was voluntary. Respondents were informed of the academic purpose of the study and assured that their answers would remain anonymous and confidential. Questionnaires were self-administered, and assistance was limited to procedural clarification. A total of 360 questionnaires were distributed. Twenty-three incomplete or otherwise invalid questionnaires were excluded during data screening, leaving 337 usable cases and a usable questionnaire rate of 93.61%. Informal refusals before questionnaire distribution were not recorded separately; accordingly, this percentage represents the proportion of distributed questionnaires retained for analysis rather than a population response rate. The final sample was comparable with those used in related latent-variable studies in hospitality research (Li et al., 2025). The convenience procedure constrained population representativeness. Participation may have been more attractive to guests with stronger environmental interests or greater willingness to complete a sustainability survey. The sample also contained comparatively high proportions of women, younger adults, and students. Recruitment from hotels that publicly communicated environmental practices may additionally have restricted variation in sustainability exposure. These characteristics delimited the interpretation of the results to the sampled guests and accommodation settings.

### 3.4 Measurement development and adaptation

Measurement development proceeded from established multi-item scales in tourism and hospitality research. Items were selected according to their conceptual correspondence with the focal constructs and adapted to rural hotel and within-stay contexts. Responses were recorded on seven-point Likert-type scales ranging from 1 (“strongly disagree”) to 7 (“strongly agree”), consistent with related hospitality measurement practices (Manosuthi et al., 2026). Full item wording is reported in Supplementary Table S2, and construct definitions are provided in Supplementary Table S3. Perceived sustainability authenticity captured whether hotel environmental practices appeared genuine, credible, transparent, consistent, and embedded in routine operations (Chua et al., 2024; Moon et al., 2023; Yu et al., 2024). Moral emotions captured moral inspiration, pride, and affective engagement associated with environmental responsibility (Lin et al., 2023; Tran et al., 2025; Zhang et al., 2025). Psychological ownership represented a temporary personal connection, concern, and perceived responsibility toward the hotel and its environment (Chen and Wu, 2024; Li et al., 2021; Yoon et al., 2025). Responsible environmental cooperation comprised voluntary compliance, additional effort, encouragement of other guests, constructive support, and willingness to accept minor inconveniences (Liu et al., 2025; Thai and Nguyen, 2022). Green skepticism captured respondents’ tendency to question the sincerity, credibility, and environmental value of hotel sustainability claims (Yoon and Chen, 2017; Zhang et al., 2021). Where source items were available in English, they were translated into Chinese by a bilingual researcher and independently back-translated by another bilingual scholar. The research team compared the source and back-translated versions and reconciled discrepancies to preserve conceptual equivalence rather than literal wording. Researchers familiar with tourism, hospitality, and environmental behavior subsequently reviewed the adapted instrument for construct correspondence and contextual appropriateness. The questionnaire was then pretested with a small group of hotel guests to assess clarity, readability, and relevance to the accommodation setting. Feedback from this assessment informed minor wording revisions before the main survey.

### 3.5 Assessment of common-method variance

Because the variables were collected through one self-reported questionnaire, common-method variance remained a potential source of covariance. Procedural safeguards included respondent anonymity, neutral item wording, reduced evaluation apprehension, and mixed presentation of conceptually distinct measures. Harman’s single-factor test was used as an initial diagnostic, and a one-factor confirmatory model was compared with the proposed multidimensional model. These procedures were interpreted cautiously because neither can exclude common-method variance (Tandon et al., 2023). The diagnostics did not indicate that one general factor dominated the covariance structure; nevertheless, common-method variance arising from the single-source design could not be ruled out.

### 3.6 Structural equation modelling and indirect associations

Data screening and descriptive analyses were conducted in IBM SPSS Statistics 29. The measurement and structural models were estimated through CB-SEM in IBM SPSS Amos 29 using maximum-likelihood estimation. Questionnaires with substantial missing or invalid responses were removed before analysis; the retained dataset comprised complete usable cases. Distributional properties were examined during preliminary data screening, and bias-corrected bootstrapping was used for indirect associations to reduce reliance on the normality assumption for their sampling distributions. Each latent construct was identified by fixing its first indicator loading to 1.0, which accounts for the absence of a t-value for the reference indicator. Measurement adequacy was evaluated using standardized loadings, Cronbach’s alpha, composite reliability, and average variance extracted. Discriminant validity was examined using the Fornell–Larcker criterion. Global model fit was assessed using χ^2^/df, the comparative fit index, the Tucker–Lewis index, the root mean square error of approximation, and the standardized root mean square residual. The structural model subsequently estimated the hypothesized direct associations. Indirect associations were assessed using 5,000 bootstrap resamples and 95% bias-corrected confidence intervals. An indirect association was regarded as statistically supported when its confidence interval excluded zero (Zhao et al., 2025).

### 3.7 Conditional indirect-association analysis

Green skepticism was specified as a first-stage moderator of the associations between perceived sustainability authenticity and each mediator. Conditional process analyses were conducted in IBM SPSS Statistics 29 using the PROCESS macro, Model 7, with moral emotions and psychological ownership examined as parallel conditional pathways. Composite scores for perceived sustainability, authenticity, and green skepticism were mean-centered before their product terms were calculated. The interaction terms were entered into the equations predicting moral emotions and psychological ownership; green skepticism was not specified as a moderator of the direct association with responsible environmental cooperation or of the mediator–outcome associations. Conditional indirect associations were estimated at the low (−1 SD), mean, and high (+1 SD) levels of green skepticism using 5,000 bootstrap resamples and 95% bias-corrected confidence intervals. An index of moderated mediation was calculated for each indirect pathway. Conditional variation was regarded as statistically supported when the confidence interval for the corresponding index excluded zero. The analysis quantified whether indirect associations differed across levels of skepticism; it did not establish that skepticism altered a causal or temporal psychological process.

## 4 Results

### 4.1 Sample characteristics

The final analytical sample comprised 337 guests staying in rural hotels in Yangshuo. Women represented 66.47% of respondents (*n* = 224), while men represented 33.53% (*n* = 113). The largest age groups were 20–29 years (43.03%) and 30–39 years (25.52%). Company employees constituted 34.12% of the sample, students 28.78%, and freelance or self-employed respondents 22.26%. Most participants held a college or university degree (70.04%), whereas 15.13% held a graduate degree. Respondents most frequently reported stays of 2 days (48.37%) or 3 days (25.82%). The “1 day” category represented one completed overnight stay. Full sample characteristics are reported in Table 1.

**Table 1:** Demographic profile of respondents (*N* = 337).

Variable	Category	Frequency	Percentage (%)
Gender	Male	113	33.53
	Female	224	66.47
Age (years)	Under 20	26	7.72
	20–29	145	43.03
	30–39	86	25.52
	40–49	46	13.65
	50–59	23	6.82
	60 or above	11	3.26
Occupation	Civil servant/public institution employee	29	8.61
	Company employee	115	34.12
	Student	97	28.78
	Freelancer/self-employed	75	22.26
	Retired	11	3.26
	Other	10	2.97
Education level	Junior high school or below	5	1.48
	High school or vocational school	45	13.35
	College or university degree	236	70.04
	Graduate degree	51	15.13
Monthly income (RMB)	2,000 or below	60	17.80
	2,001–4,000	121	35.91
	4,001–6,000	78	23.15
	6,001–8,000	38	11.28
	8,001–10,000	23	6.82
	10,001 or above	17	5.04
Length of stay	1 day	70	20.77
	2 days	163	48.37
	3 days	87	25.82
	4 days or more	17	5.04

### 4.2 Preliminary and descriptive results

Item-aggregated construct means were calculated from the item means reported in Table 2. The mean scores were 5.119 for perceived sustainability authenticity, 5.362 for moral emotions, 5.083 for psychological ownership, 5.519 for responsible environmental behavior, and 5.415 for green skepticism. Perceived sustainability authenticity was positively correlated with moral emotions (*r* = 0.53, *p* < 0.001), psychological ownership (*r* = 0.49, *p* < 0.001) and responsible environmental cooperation (*r* = 0.51, *p* < 0.001). Moral emotions were positively correlated with psychological ownership (*r* = 0.56, *p* < 0.001) and responsible environmental cooperation (*r* = 0.58, *p* < 0.001), whereas psychological ownership was positively correlated with responsible environmental cooperation (*r* = 0.55, *p* < 0.001) (Table 3). Green skepticism showed smaller positive correlations with perceived sustainability authenticity (*r* = 0.21, *p* < 0.01), moral emotions (*r* = 0.19, *p* < 0.01), psychological ownership (*r* = 0.17, *p* < 0.05), and responsible environmental cooperation (*r* = 0.23, *p* < 0.01). These bivariate associations were consistent with the directions proposed in the statistical model.

**Table 2:** Measurement model results: confirmatory factor analysis, reliability, and convergence validity.

Construct/item	Mean	t-value	Standardized loading	Cronbach’s α	CR	AVE
Perceived sustainability authenticity (PSA)	5.119			0.935	0.937	0.576
PSA1	5.15		0.790			
PSA2	4.91	14.370	0.724			
PSA3	5.21	16.597	0.810			
PSA4	5.20	16.247	0.797			
PSA5	5.18	14.410	0.725			
PSA6	5.35	15.521	0.769			
PSA7	5.23	16.587	0.809			
PSA8	5.20	15.296	0.760			
PSA9	5.17	15.497	0.768			
PSA10	4.85	14.127	0.714			
PSA11	4.87	13.117	0.672			
Moral emotions (ME)	5.362			0.877	0.877	0.641
ME1	5.34		0.795			
ME2	5.34	15.565	0.812			
ME3	5.52	14.958	0.783			
ME4	5.25	15.598	0.813			
Psychological ownership (PO)	5.083			0.864	0.871	0.576
PO1	5.20		0.760			
PO2	5.15	13.995	0.773			
PO3	5.11	12.277	0.684			
PO4	5.01	14.747	0.814			
PO5	4.94	13.728	0.759			
Responsible environmental cooperation (REC)	5.519			0.874	0.881	0.597
RTB1	5.78		0.844			
RTB2	5.76	15.916	0.771			
RTB3	5.89	16.072	0.776			
RTB4	5.15	15.569	0.758			
RTB5	5.01	14.263	0.711			
Green skepticism (GS)	5.415			0.917	0.919	0.657
GS1	5.44		0.849			
GS2	5.36	19.352	0.844			
GS3	5.53	17.511	0.793			
GS4	5.29	17.649	0.797			
GS5	5.63	19.223	0.841			
GS6	5.25	15.537	0.732			

The abbreviations used are composite reliability (CR) and average variance extracted (AVE).

**Table 3:** Descriptive statistics and correlations.

Construct	Mean	SD	1	2	3	4	5
1. Perceived sustainability authenticity (PSA)	5.12	0.89	1				
2. Moral emotions (ME)	5.36	0.92	0.53***	1			
3. Psychological ownership (PO)	5.08	0.87	0.49***	0.56***	1		
4. Responsible environmental cooperation (REC)	5.52	0.91	0.51***	0.58***	0.55***	1	
5. Green skepticism (GS)	5.42	1.03	0.21**	0.19**	0.17*	0.23**	1

*p < 0.05; **p < 0.01; ***p < 0.001.

### 4.3 Measurement model

Confirmatory factor analysis was used to assess the measurement model. Standardized factor loadings ranged from 0.672 to 0.849. The first loading for each latent construct was fixed to 1.0 to establish the measurement scale; consequently, no t-value was reported for the reference indicator. All freely estimated loadings had t-values above 1.96. Internal consistency estimates were satisfactory. Cronbach’s alpha ranged from 0.864 to 0.935, and composite reliability ranged from 0.871 to 0.937. Average variance extracted ranged from 0.576 to 0.657. The results were therefore consistent with acceptable internal consistency and convergent validity. Full item loadings and reliability estimates are reported in Table 2.

### 4.4 Discriminant validity

Discriminant validity was assessed using the Fornell–Larcker criterion. Table 4 reports latent-factor correlations derived from the confirmatory factor model, rather than the observed composite-score correlations reported in Table 3. The square root of the AVE for each construct exceeded its latent correlations with the other constructs. For perceived sustainability authenticity, for example, the square root of the AVE was 0.759, compared with latent correlations of 0.325 with moral emotions, 0.302 with psychological ownership, 0.300 with responsible environmental cooperation, and 0.335 with green skepticism. The corresponding comparisons were also satisfied for the remaining constructs. The results were consistent with discriminant validity under the Fornell–Larcker criterion.

**Table 4:** Discriminant validity assessment (Fornell–Larcker criterion).

Construct	1	2	3	4	5
1. Perceived sustainability authenticity (PSA)	**0.759**				
2. Moral emotions (ME)	0.325	**0.800**			
3. Psychological ownership (PO)	0.302	0.317	**0.759**		
4. Responsible environmental cooperation (REC)	0.300	0.335	0.308	**0.773**	
5. Green skepticism (GS)	0.335	0.222	0.316	0.488	**0.810**

Diagonal elements (in bold) represent the square roots of the average variance extracted (AVE) for each construct. Off-diagonal elements represent Pearson correlation coefficients between constructs.

### 4.5 Structural associations

The structural model showed acceptable fit to the data: χ^2^/df = 2.41, CFI = 0.94, TLI = 0.93, RMSEA = 0.064, and SRMR = 0.041. Perceived sustainability authenticity was positively associated with responsible environmental cooperation (*β* = 0.31, *t* = 6.42, *p* < 0.001), consistent with *H1*. It was also positively associated with moral emotions (*β* = 0.53, *t* = 9.87, *p* < 0.001) and psychological ownership (*β* = 0.47, t = 8.91, *p* < 0.001), consistent with *H2* and *H5*, respectively. Moral emotions were positively associated with responsible environmental cooperation (*β* = 0.29, *t* = 5.76, *p* < 0.001), consistent with *H3*. Psychological ownership was likewise positively associated with responsible environmental cooperation (*β* = 0.27, *t* = 5.18, *p* < 0.001), consistent with *H6*. The model statistically accounted for 28% of the variance in moral emotions, 22% in psychological ownership, and 46% in responsible environmental cooperation. Table 5 reports the complete structural estimates.

**Table 5:** Structural model results and hypothesis testing.

Hypothesis	Structural path	Standardized estimate	SE	t-value	95% CI	Result
H1	PSA → RTB	0.31***	0.05	6.42	[0.21, 0.41]	Supported
H2	PSA → ME	0.53***	0.05	9.87	[0.43, 0.64]	Supported
H3	ME → RTB	0.29***	0.05	5.76	[0.19, 0.40]	Supported
H5	PSA → PO	0.47***	0.05	8.91	[0.37, 0.58]	Supported
H6	PO → RTB	0.27***	0.05	5.18	[0.16, 0.37]	Supported

Standardized coefficients are reported; ***p < 0.001; Model fit indices: χ^2^/df = 2.41; CFI = 0.94; TLI = 0.93; RMSEA = 0.064; SRMR = 0.041; Explained variance (R^2^): Moral emotions (ME) = 0.28; Psychological ownership (PO) = 0.22; Responsible environmental cooperation (REC) = 0.46.

### 4.6 Indirect associations

Bootstrapping with 5,000 resamples was used to estimate indirect associations. The indirect association between perceived sustainability authenticity and responsible environmental cooperation through moral emotions was *β* = 0.15 (SE = 0.04, 95% bias-corrected CI [0.08, 0.24]). Because the confidence interval excluded zero, the result was consistent with *H4*. The indirect association through psychological ownership was *β* = 0.13 (SE = 0.03, 95% bias-corrected CI [0.07, 0.21]), consistent with *H7*. The direct association remained statistically significant in both pathway-specific models (*β* = 0.31, SE = 0.05, 95% CI [0.21, 0.41]). The pattern was therefore consistent with partial statistical mediation, although the cross-sectional analysis did not establish temporal or causal mediation. Moral emotions accounted for 32.6% of the corresponding total association, and psychological ownership accounted for 29.5%. Table 6 reports the direct, indirect, and total associations.

**Table 6:** Mediation analysis results (bootstrapping).

Mediator	Effect type	Standardized effect	SE	95% bias-corrected CI	Result
Moral emotions (ME)	Indirect (PSA → ME → RTB)	0.15***	0.04	[0.08, 0.24]	Supported
	Direct (PSA → RTB)	0.31***	0.05	[0.21, 0.41]	
	Total Effect	0.46***	0.06	[0.34, 0.58]	
Psychological ownership (PO)	Indirect (PSA → PO → RTB)	0.13***	0.03	[0.07, 0.21]	Supported
	Direct (PSA → RTB)	0.31***	0.05	[0.21, 0.41]	
	Total effect	0.44***	0.06	[0.32, 0.56]	

Variance accounted for (VAF): Moral emotions = 32.6%; Psychological ownership = 29.5%, indicating partial mediation. ***p < 0.001.

### 4.7 Moderation and conditional indirect associations

Green skepticism was specified as a first-stage moderator of the associations between perceived sustainability authenticity and the two mediators. It was not specified as a moderator of the direct perceived sustainability–authenticity–cooperation association or of the mediator–cooperation associations. For moral emotions, the conditional indirect association was 0.09 at low green skepticism (SE = 0.03, 95% CI [0.03, 0.16]), 0.14 at the mean (SE = 0.04, 95% CI [0.06, 0.22]), and 0.21 at high green skepticism (SE = 0.05, 95% CI [0.12, 0.31]). The index of moderated mediation was 0.06 (SE = 0.02, 95% CI [0.02, 0.11]), consistent with *H8a*. For psychological ownership, the corresponding conditional indirect associations were 0.07 (SE = 0.03, 95% CI [0.02, 0.15]), 0.12 (SE = 0.04, 95% CI [0.05, 0.20]) and 0.19 (SE = 0.05, 95% CI [0.10, 0.29]). The index of moderated mediation was 0.05 (SE = 0.02, 95% CI [0.01, 0.10]), consistent with *H8b*. Thus, both indirect associations were larger at higher levels of green skepticism (Table 7). These estimates reflected conditional statistical associations rather than causal changes in psychological processes.

**Table 7:** Moderated mediation analysis results (conditional indirect effects of green skepticism).

Mediator	Green skepticism level	Indirect effect (PSA → mediator → RTB)	SE	95% bias-Corrected CI
Panel A. Conditional indirect effects at different levels of green skepticism				
Moral emotions (ME)	Low (−1 SD)	0.09*	0.03	[0.03, 0.16]
	Mean	0.14**	0.04	[0.06, 0.22]
	High (+1 SD)	0.21***	0.05	[0.12, 0.31]
Psychological ownership (PO)	Low (−1 SD)	0.07*	0.03	[0.02, 0.15]
	Mean	0.12**	0.04	[0.05, 0.20]
	High (+1 SD)	0.19***	0.05	[0.10, 0.29]

Moderated mediation path	Index	SE	95% bias-corrected CI	Result
Panel B. Index of moderated mediation				
PSA → ME → RTB × GS	0.06	0.02	[0.02, 0.11]	Supported
PSA → PO → RTB × GS	0.05	0.02	[0.01, 0.10]	Supported

*p < 0.05; **p < 0.01; ***p < 0.001.

## 5 Discussion

The study examined how perceived sustainability authenticity was associated with guests’ self-reported responsible environmental cooperation, whether moral emotions and psychological ownership statistically accounted for distinct indirect associations, and whether these associations varied across levels of green skepticism. Guests reported stronger environmental cooperation when hotel sustainability practices appeared genuine, credible, and embedded in their routine operations. This pattern was consistent with prior evidence connecting credible environmental practices with supportive guest responses while extending that literature from general engagement and evaluation to discretionary within-stay cooperation (He and Zaman, 2024; Liu and Nguyen Hoang Thanh, 2025; Yu et al., 2024). The association did not establish that perceived authenticity precedes cooperation. Guests who were already environmentally engaged may also have evaluated hotel practices more favorably, and shared-method covariance may have contributed to the observed relationship. Moral emotions and psychological ownership each statistically accounted for a positive indirect association between perceived sustainability authenticity and responsible environmental cooperation. Moral emotions represented an affective and norm-responsive pathway. Guests who regarded environmental practices as authentic also reported stronger inspiration, pride, and ethically grounded engagement, which corresponded with greater cooperation (Meng et al., 2022; Tran et al., 2025). This finding complemented research that centered on attitudes, perceived value, and behavioral intentions by indicating that affective evaluations were also relevant to discretionary environmental conduct (Fu et al., 2025; Sharma et al., 2025; Srivastava et al., 2024). In shared tourism settings, such emotions may be particularly salient because environmental outcomes depend on the aggregated conduct of multiple participants (Li et al., 2026; Wang et al., 2023; Zhang et al., 2025).

Psychological ownership represented a distinct responsibility-oriented pathway. Guests who perceived sustainability practices as authentic reported stronger personal connection and responsibility for the hotel and its surroundings. These perceptions were positively associated with environmental cooperation. This result was consistent with research linking psychological ownership with stewardship and supportive conduct in tourism and service settings (Li et al., 2021; Wu et al., 2025; Yoon et al., 2025). In the present study, psychological ownership referred to a temporary sense that the accommodation and its environmental condition were partly “mine” or “my responsibility,” rather than to a durable attachment. Such a stay-level orientation may correspond with the willingness to protect shared resources and accept minor inconveniences during the visit (Gong et al., 2025). The two indirect associations were complementary but did not establish independent psychological mechanisms. Moral emotions captured affective responses to perceived ethical conduct, whereas psychological ownership captured self-referential connections and responsibility. Their simultaneous statistical significance indicated that each retained a distinct association within the parallel model. It did not establish that they occurred separately, simultaneously, or in the proposed temporal order.

Both indirect associations were larger at higher levels of green skepticism. This pattern differed from a simple view of skepticism as uniformly associated with rejection of environmental claims (Yoon and Chen, 2017; Zhang et al., 2021). Skeptical guests may have applied more demanding credibility standards when comparing sustainability communication with observable practices. Where practices appeared consistent and operationally embedded, perceived authenticity may have become more diagnostically relevant to their emotional and responsibility-oriented evaluations (Bakari et al., 2024; Rahman et al., 2015; Zhao et al., 2025). This interpretation remained conditional: the analysis identified differences in statistical associations across skepticism levels but did not establish that skepticism altered a causal psychological process.

### 5.1 Theoretical implications

The study offers four bounded theoretical implications. First, perceived sustainability authenticity was positioned as a contextual credibility cue rather than only as a brand-related, marketing, or corporate-responsibility evaluation. Existing research has commonly examined sustainability initiatives in relation to organizational reputation, consumer evaluation, and strategic positioning (El Baroudi et al., 2023; Gupta and Arora, 2024; Truong et al., 2025). The present findings indicated that guests’ assessments of whether environmental claims corresponded with routine operations were also associated with their reported conduct during the service encounter. Second, the study differentiated two indirect pathways within one model. Moral emotions captured affective and normative meaning, whereas psychological ownership captured personal connection and perceived responsibility. This integration extended prior work on green authenticity and responsible tourist conduct without presenting either construct as novel (Shishan et al., 2025; Yu et al., 2025). The parallel specification clarified conceptual differentiation but did not verify temporal ordering or psychological independence. Third, green skepticism was interpreted as a conditional credibility filter. The findings indicated that critical evaluation was not necessarily incompatible with environmental cooperation. Instead, authenticity appeared more consequential when guests applied stricter standards to environmental claims. Fourth, soft behavioral governance provided a bounded interpretive lens for these relationships. The lens directed attention to legitimacy, contextual consistency, and voluntary alignment rather than formal regulation or sanctions. It did not constitute a new governance theory or establish self-regulation as a causal process (Baah and Kim, 2025; Srivastava et al., 2025; Zhao et al., 2023).

### 5.2 Managerial implications

Accommodation managers may derive value from making environmental practices visible, internally consistent, and verifiable. Sustainability communication can be aligned with routine operations, staff conduct, and physical service arrangements so that guests can compare claims with observable evidence. Specific information concerning practices, procedures, and progress may be more credible than broad environmental assertions, particularly among skeptical guests (Alyahia et al., 2024; Perano et al., 2025; Pham et al., 2025). Guest participation may also be invited through low-burden arrangements that clarify the relevance of requested conduct without relying on guilt or exaggerated claims. Examples include accessible waste-separation facilities, clear resource-conservation information, voluntary feedback channels, and opportunities to participate in place-specific environmental activities. Such arrangements may support temporary stewardship by making environmental responsibility personally relevant during the stay. At the destination level, coordination across accommodation providers, infrastructure, and visitor services may improve the consistency of environmental cues in nature-dependent settings (Deng et al., 2025). These recommendations concern credibility and participation; the study did not verify reductions in waste, emissions, energy use, or water consumption.

### 5.3 Limitations and future research

Several limitations bounded the findings. The cross-sectional design did not establish temporal ordering, causal mediation, or persistence after departure. Longitudinal studies, repeated measurements, and field experiments could examine whether authenticity perceptions preceded changes in emotions, ownership, and cooperation over time (Arif and Yu, 2026). The single-source, self-reported design also remained vulnerable to common-method variance and socially desirable responses. Future research could combine temporally separated surveys with behavioral observation, hotel records, resource-use indicators, or multi-source assessments. Convenience sampling introduced self-selection, and the sample contained demographic imbalances in gender, age, and student representation. Stratified or probability-based recruitment across broader visitor groups would provide stronger population coverage. The study was also restricted to selected rural hotels in one destination and one fieldwork period. Multi-destination and cross-national designs could examine whether the associations varied across urban, resort, and nature-based contexts, as well as across seasons and certification regimes. Finally, the model did not assess alternative ordering among the focal constructs, prior environmental orientation, repeated visitation, or durable place attachment. Competing longitudinal models could compare parallel, sequential, and reciprocal specifications. Research could also examine whether temporary psychological ownership persisted after departure and whether reported cooperation corresponded with objectively recorded environmental conduct (Orea-Giner et al., 2025).

## 6 Conclusion

This study examined the association between perceived sustainability authenticity and self-reported responsible environmental cooperation in nature-based accommodation. Guests reported stronger within-stay cooperation when environmental practices appeared genuine, credible, transparent, and embedded in routine hotel operations. Perceived sustainability authenticity was therefore positioned as a contextual credibility cue rather than solely as a promotional or reputational evaluation. Moral emotions and psychological ownership each statistically accounted for a positive indirect association between perceived sustainability authenticity and responsible environmental cooperation. Moral emotions reflected the affective and normative significance assigned to credible environmental practices, whereas psychological ownership reflected a temporary sense of personal connection and responsibility toward the accommodation and its environment. Their parallel significance indicated conceptually differentiated associations but did not establish statistical independence, temporal ordering, or causal mediation. Both indirect associations were larger at higher levels of green skepticism. This pattern was consistent with the interpretation of skepticism as a conditional credibility filter, whereby perceived authenticity was more strongly associated with moral-emotional and responsibility-oriented responses among guests who evaluated environmental claims more critically. Taken together, the findings linked perceived sustainability authenticity with voluntary within-stay environmental cooperation through complementary affective and self-referential associations. Because the evidence was cross-sectional, single-source, and self-reported, it did not establish temporal ordering, objectively verified environmental conduct, causal mediation, or persistence beyond the accommodation stay.

## Data Availability

The raw data supporting the conclusions of this article will be made available by the authors, without undue reservation.
