# Supplementary material for: Perceived sustainability authenticity as a place-based psychological cue: moral emotions, psychological ownership, and responsible environmental cooperation in nature-based accommodation
Source: Front Psychol. 2026 Jul 14;17:1903093. doi: 10.3389/fpsyg.2026.1903093 (PMC13407275; doi:10.3389/fpsyg.2026.1903093)
Supplement: Supplementary file 1 [file Table_1.docx]

Supplementary Material

**Table S1.** Positioning of the present study relative to closely related literature.

| Study | Context | Main constructs / focus | Key outcome(s) examined | What this study established | Remaining gap | Contribution of the present study |
| --- | --- | --- | --- | --- | --- | --- |
| Moon et al. (2023) | Green hospitality/ tourism marketing | Green brand authenticity, warm glow, green satisfaction, willingness to pay more | Consumer evaluation and willingness-to-pay responses | Showed that authenticity matters for favorable green consumption responses | Focused mainly on attitudinal /evaluative outcomes rather than discretionary pro-environmental behavior | Extends authenticity research from evaluative outcomes to responsible tourist behavior, and explains the process through moral emotions and psychological ownership |
| Chua et al. (2024) | Hospitality brands | Green authenticity, stereotypes, customer–green brand relationships | Relationship-building outcomes | Demonstrated that authenticity shapes green brand relationships in hospitality | Did not explain how authenticity translates into tourists’ responsible environmental conduct during the stay | Moves beyond brand relationship outcomes by linking perceived sustainability authenticity to within-stay responsible tourist behavior |
| Yu et al. (2024) | Green hospitality brands | Green authenticity, well-being, customer engagement, approach behavior | Engagement and approach behavior | Showed that authenticity can improve customer engagement and approach responses | Behavioral outcomes were not centered on tourists’ voluntary environmental cooperation | Repositions authenticity as a cue associated with discretionary environmental cooperation, not only engagement or approach behavior |
| Thai and Nguyen (2022) | Green hotels | Green hotel practices, green hotel image, customer satisfaction, customer citizenship behavior | Extra-role/customer citizenship behavior | Established that green practices can stimulate extra-role behavior through cognitive/attitudinal routes | Did not examine authenticity as a central explanatory cue, nor affective and ownership-based mediators together | Adds perceived sustainability authenticity as the focal antecedent and integrates moral emotions and psychological ownership in one model |
| Liu et al. (2025) | Green hotels (cross-cultural) | Green hotel practices, in-role and extra-role behavior | Customer in-role and extra-role behavior | Demonstrated that green practices are associated with customer behavioral responses | Focused on practices broadly, not on whether those practices are perceived as authentic | Clarifies that the perceived authenticity of sustainability practices matters, not merely the existence of such practices |
| Tran et al. (2025) | Green hotels | Green hotel practices, pride, moral elevation, customer citizenship intentions | Green customer citizenship behavioral intentions | Highlighted the importance of moral emotions in green behavior processes | Focused on moral-emotion pathways, without integrating authenticity and ownership together | Shows that moral emotions are one complementary mechanism through which authenticity may relate to responsible tourist behavior |
| Zhang et al. (2025) | Rural tourism | Moral gaze, pro-environmental behavior | Pro-environmental behavior | Confirmed that ethically salient cues can stimulate pro-environmental responses | Did not examine sustainability authenticity or ownership-based internalization | Extends the moral-emotion literature by linking authentic sustainability signals to behavior and pairing moral emotions with psychological ownership |
| Li et al. (2021) | Hotel customers | Antecedents and consequences of psychological ownership | Behavioral and relational outcomes | Established that psychological ownership can influence customer responses in hospitality | Did not examine sustainability authenticity or pro-environmental tourist behavior | Applies psychological ownership to the sustainability domain and interprets it as a responsibility-oriented mechanism associated with responsible tourist behavior |
| Chen and Wu (2024) | Cultural tourism | Psychological ownership and sustainable behavior | Tourists’ sustainable behavior | Showed that psychological ownership is relevant to sustainable tourist behavior | Did not connect ownership to authenticity cues or compare it with emotion-based pathways | Extends this stream by examining how perceived sustainability authenticity may activate psychological ownership, alongside moral emotions |
| Rahman et al. (2015) | Hotels/greenwashing context | Consumer reactions to green initiatives / greenwashing | Consumer responses to green claims | Showed that questionable green claims can trigger negative reactions | Focused on consequences of greenwashing, not on when authenticity becomes more behaviorally consequential | Helps motivate our boundary-condition argument that authenticity may matter more when tourists evaluate claims critically |
| Yoon and Chen (2017) | Hotel customers | Environmental knowledge, concern, green marketing skepticism, behavioral intentions | Behavioral intentions | Established skepticism as an important interpretive orientation in green hospitality contexts | Did not test skepticism as a moderator of authenticity-based behavioral mechanisms | Extends skepticism research by modeling green skepticism as a moderator of the indirect effects of authenticity |
| Zhang et al. (2021) | Restaurant green demarketing | Green skepticism, benefit associations, green reputation, intentions | Dining intentions | Showed that skepticism shapes responses to green marketing claims | Not situated in tourism-stay sustainability behavior, and not integrated with authenticity, emotions, and ownership | Broadens skepticism research into a tourism sustainability setting and links it to conditional indirect effects |

**Table S2.** Measurement items for study constructs.

| Variables (codes) | Measurement statement |
| --- | --- |
| Perceived Sustainability Authenticity (PSA); Chua et al., 2024; Moon et al., 2023; Yu et al., 2024 | |
| PSA1 | This hotel’s environmental practices feel genuine, not staged for marketing. |
| PSA2 | The hotel’s sustainability claims are truthful and believable. |
| PSA3 | The hotel’s green practices are consistent across different areas (e.g., rooms, public spaces, services). |
| PSA4 | The hotel’s environmental actions are integrated into its daily operations, not occasional campaigns. |
| PSA5 | The hotel provides clear and concrete information about how it reduces environmental impact. |
| PSA6 | The hotel’s green efforts are substantial rather than symbolic gestures. |
| PSA7 | The hotel’s environmental initiatives match what I actually observe during my stay. |
| PSA8 | The hotel appears willing to invest in sustainability even if it increases costs or effort. |
| PSA9 | The hotel’s sustainability communication is transparent and not exaggerated. |
| PSA10 | The hotel’s environmental performance seems measurable (e.g., targets, outcomes, or evidence). |
| PSA11 | Overall, I trust that this hotel is sincerely committed to environmental protection. |
| Moral Emotions (ME); Lin et al., 2023; Tran et al., 2025; Zhang et al., 2025 | |
| ME1 | Seeing this hotel’s environmental practices makes me feel morally inspired to act responsibly. |
| ME2 | I feel a sense of pride when I think about supporting this hotel’s environmental efforts. |
| ME3 | This hotel’s sustainability practices make me feel that doing the right thing is valued here. |
| ME4 | Observing this hotel’s environmental actions motivates me emotionally to behave in an environmentally responsible way. |
| Psychological Ownership (PO); Chen & Wu, 2024; Li et al., 2021; Yoon et al., 2025 | |
| PO1 | I feel a personal sense of ownership toward this hotel. |
| PO2 | This hotel feels like “my place” during my stay. |
| PO3 | I feel personally connected to this hotel. |
| PO4 | I feel a sense of responsibility for taking care of this hotel and its environment. |
| PO5 | If something harmful happened to this hotel or its surroundings, I would feel personally concerned. |
| Responsible Tourist Behavior (RTB); Liu et al., 2025; Thai & Nguyen, 2022; Tran et al., 2025 | |
| RTB1 | I willingly follow the hotel’s environmental practices even when they are not required. |
| RTB2 | I am willing to make extra effort to support this hotel’s environmental initiatives. |
| RTB3 | I would encourage other guests to behave in an environmentally responsible way at this hotel. |
| RTB4 | I would support or suggest ideas to help this hotel improve its environmental performance. |
| RTB5 | I am willing to accept minor inconvenience for the sake of this hotel’s environmental goals. |
| Green Skepticism (GS); Rahman et al., 2015; Yoon & Chen, 2017; Zhang et al., 2021 | |
| GS1 | I am often skeptical about the environmental claims made by hotels. |
| GS2 | I suspect that some hotel sustainability initiatives are mainly for marketing purposes. |
| GS3 | I question whether hotels’ green practices truly reduce environmental impact. |
| GS4 | I believe that hotels sometimes exaggerate their environmental performance. |
| GS5 | I am cautious about trusting hotels’ sustainability messages without clear evidence. |
| GS6 | When hotels promote themselves as “green,” I tend to doubt how genuine those claims are. |

**Table S3.** Definitions of study constructs.

| Construct | Definition |
| --- | --- |
| Perceived Sustainability Authenticity (PSA) | Perceived sustainability authenticity refers to stakeholders’ evaluation that an organization’s sustainability initiatives and environmental claims are genuine, substantive, and embedded within its operational practices rather than symbolic or primarily promotional. It reflects the extent to which sustainability efforts are perceived as consistent, transparent, credible, and congruent with the organization’s actual practices and stakeholder experiences. |
| Moral Emotions (ME) | Moral emotions are positively conceived, ethics-based affective responses, such as moral inspiration, pride, and moral elevation, produced when customers observe a hotel’s responsible environmental practices. These emotions promote behavioral alignment with moral norms, strengthening environmentally responsible actions beyond instrumental self-interest. |
| Psychological Ownership (PO) | Psychological ownership is a psychological state in which customers experience a sense of personal possession, emotional attachment, and responsibility toward a hotel or its environment, despite the absence of legal ownership. In hospitality contexts, it reflects the feeling that the place is “mine”, motivating customers to care for, protect, and act in the interest of the hotel and its surrounding environment. |
| Responsible Tourist Behavior (RTB) | Responsible tourist behavior refers to tourists’ voluntary actions that aim to minimize negative environmental and social impacts while contributing positively to the sustainability of tourism destinations. It reflects tourists’ willingness to act in accordance with environmental norms, conservation principles, and destination stewardship during their travel experience. |
| Green Skepticism (GS) | Green skepticism is a consumer’s tendency to question the credibility, sincerity, or effectiveness of hotels’ environmental claims and sustainability initiatives. It reflects a critical evaluative stance in which customers doubt whether green practices meaningfully reduce environmental impacts or primarily serve marketing and image management. |

**References**

Chen, W., & Wu, M. (2024). Exploring the role of psychological ownership in tourists’ shift toward sustainable behavior in cultural tourism. *Journal of Sustainable Tourism*, *32*(11), 2403–2422. https://doi.org/10.1080/09669582.2024.2341890

Chua, B.-L., Kim, S., Baah, N. G., Moon, H., Yu, J., & Han, H. (2024). When hospitality brands go green: the role of authenticity and stereotypes in building customer-green brand relationships. *Journal of Sustainable Tourism*, *32*(6), 1118–1141. https://doi.org/10.1080/09669582.2023.2203406

Li, S., Qu, H., & Wei, M. (2021). Antecedents and consequences of hotel customers’ psychological ownership. *International Journal of Hospitality Management*, *93*, 102773. https://doi.org/10.1016/j.ijhm.2020.102773

Lin, J., Zhou, Z., Zheng, F., Jiang, X., & Nguyen, N. (2023). How do hotel star ratings affect the relationship between environmental CSR and green word‐of‐mouth? *Corporate Social Responsibility and Environmental Management*, *30*(5), 2651–2663. https://doi.org/10.1002/csr.2508

Liu, C., Li, J., & Ogada, H. (2025). The impact of green hotel practices on customer in-role and extra-role behaviors: a cross-cultural perspective. *International Journal of Contemporary Hospitality Management*, *37*(12), 4084–4100. https://doi.org/10.1108/IJCHM-02-2025-0257

Moon, H., Yu, J., Chua, B.-L., & Han, H. (2023). Impact of green brand authenticity on warm glow, green satisfaction, and willingness to pay more. *Journal of Travel & Tourism Marketing*, *40*(4), 326–344. https://doi.org/10.1080/10548408.2023.2245446

Rahman, I., Park, J., & Chi, C. G.-q. (2015). Consequences of “greenwashing”: Consumers’ reactions to hotels’ green initiatives. *International Journal of Contemporary Hospitality Management*, *27*(6), 1054–1081. https://doi.org/10.1108/IJCHM-04-2014-0202

Thai, K. P., & Nguyen, Q. H. (2022). How green hotel practices stimulates customer citizenship behavior? examining the role of green hotel mage and customer satisfaction in Vietnam. *GeoJournal of Tourism and Geosites*, *40*, 274–282. https://doi.org/10.30892/gtg.40133-829

Tran, D. V., Nguyen, D. M., & Nguyen, T. (2025). Fostering green customer citizenship behavioral intentions through green hotel practices: the roles of pride, moral elevation, and hotel star ratings. *Journal of Sustainable Tourism*, *33*(1), 122–142. https://doi.org/10.1080/09669582.2024.2316296

Yoon, D., & Chen, R. J. (2017). A green shadow: the influence of hotel customers' environmental knowledge and concern on green marketing skepticism and behavioral intentions. *Tourism Analysis*, *22*(3), 281–293. https://doi.org/10.3727/108354217X14955605216032

Yoon, Y. R., Peck, J., & Shu, S. B. (2025). Increasing Hotel Loyalty Through Psychological Ownership. *Cornell Hospitality Quarterly*, 19389655241309634. https://doi.org/10.1177/19389655241309634

Yu, J., Baah, N. G., Kim, S., Moon, H., Chua, B.-L., & Han, H. (2024). Effects of green authenticity on well-being, customer engagement and approach behavior toward green hospitality brands. *International Journal of Contemporary Hospitality Management*, *36*(9), 3129–3150. https://doi.org/10.1108/IJCHM-02-2023-0194

Zhang, H., Tan, Y., Long, S., & Zhou, Q. (2025). The influencing mechanism of moral gaze in rural tourism on pro-environmental behavior. *Journal of Hospitality and Tourism Management*, *63*, 30–42. https://doi.org/10.1016/j.jhtm.2025.03.004

Zhang, X., Shao, X., Jeong, E., & Jang, S. (2021). The effects of restaurant green demarketing on green skepticism and dining intentions: Investigating the roles of benefit associations and green reputation. *International Journal of Hospitality Management*, *97*, 103007. https://doi.org/10.1016/j.ijhm.2021.103007
